# Intraoperative Ultrasound Leads to Conservative Management of Benign Ovarian Tumors: A Retrospective, Single-Center Study

**DOI:** 10.1055/s-0039-1698774

**Published:** 2019-11

**Authors:** Levon Badiglian-Filho, Ademir Narciso de Oliveira Menezes, Carlos Chaves Faloppa, Elza Mieko Fukazawa, Henrique Mantoan, Lillian Yuri Kumagai, Glauco Baiocchi

**Affiliations:** 1 Department of Gynecologic Oncology, A.C. Camargo Cancer Center, São Paulo, São Paulo, Brazil

**Keywords:** intraoperative, laparoscopy, ovary, sparing, conservative surgery, ultrasound, oophoplasty, intraoperatório, laparoscopia, ovário, preservação, cirurgia conservadora, ultrassom, ooforoplastia

## Abstract

**Objective** To evaluate whether the use of intraoperative ultrasound leads to more conservative surgeries for benign ovarian tumors.

**Methods** Women who underwent surgery between 2007 and 2017 for benign ovarian tumors were retrospectively analyzed. The women were classified into two groups: those who underwent intraoperative ultrasound (group A) and those who did not (group B). In group A, minimally-invasive surgery was performed for most patients (a specific laparoscopic ultrasound probe was used), and four patients were submitted to laparotomy (a linear ultrasound probe was used). The primary endpoint was ovarian sparing surgery (oophoroplasty).

**Results** Among the 82 cases identified, only 36 met the inclusion criteria for the present study. Out of these cases, 25 underwent intraoperative ultrasound, and 11 did not. There were no significant differences in arterial hypertension, diabetes, smoking history, and body mass index for the two groups (*p* = 0.450). Tumor diameter was also similar for both groups, ranging from 1 cm to 11 cm in group A and from 1.3 cm to 10 cm in group B (*p* = 0.594). Tumor histology confirmed mature teratomas for all of the cases in group B and for 68.0% of the cases in group A. When the intraoperative ultrasound was performed, a more conservative surgery was performed (*p* < 0.001).

**Conclusion** The use of intraoperative ultrasound resulted in more conservative surgeries for the resection of benign ovarian tumors at our center.

## Resumo

**Objetivo** Avaliar se o uso do ultrassom intraoperatório leva a cirurgias mais conservadoras para tumores ovarianos benignos.

**Métodos** Mulheres que foram submetidas a cirurgia entre 2007 e 2017 por tumores ovarianos benignos foram analisadas retrospectivamente. As mulheres foram classificadas em dois grupos: aquelas que foram submetidas ao ultrassom intraoperatório (grupo A), e aquelas que não o foram (grupo B). No grupo A, foi realizada cirurgia minimamente invasiva na maioria das pacientes (foi usada sonda ultrassonográfica laparoscópica específica), e quatro pacientes foram submetidas a laparotomia (foi usada sonda ultrassonográfica linear). O desfecho primário foi a cirurgia preservadora do ovário (ooforoplastia).

**Resultados** Entre os 82 casos identificados, somente 36 atenderam aos critérios de inclusão para este estudo. Destes, 25 pacientes foram submetidas ao ultrassom intraoperatório, e 11 não o foram. Não houve diferenças significantes em relação à pressão arterial, diabetes, tabagismo e índice de massa corporal entre os dois grupos (*p* = 0.450). O diâmetro do tumor também foi similar entre os dois grupos, variando de 1 cm a 11cm no grupo A, e de 1,3 cm a 10 cm no grupo B (*p* = 0.594). A histologia dos tumores confirmou teratoma maduro para todos os casos do grupo B, e para 68,0% dos casos do grupo A. Mais cirurgias conservadoras foram realizadas quando o ultrassom intraoperatório foi realizado (*p* < 0.001).

**Conclusão** O uso do ultrassom intraoperatório resultou em mais cirurgias conservadoras na ressecção de tumores benignos do ovário em nossa instituição.

## Introduction

Benign ovarian tumors are commonly diagnosed, and most are managed with periodic imaging exams, such as transvaginal ultrasound or magnetic resonance, rather than surgery.^1^ However, when malignancy is suspected due to patient cancer phobia, family history, presence of a growing nodule or cyst, and other reasons, surgical management may be necessary. Many of the patients who undergo surgery are postmenopausal women, and there is no concern for ovarian preservation. However, for patients who are of reproductive age, sparing of the ovarian parenchyma is desirable. Approximately 13% of ovarian cancer patients are younger than 45 years of age.^2^ ^3^ ^4^ ^5^

The detection of normal ovarian parenchyma can be challenging. A surgeon can evaluate an ovary visually, by palpation, or by intraoperative ultrasound. The risk of malignancy must also be considered, since performing oophoroplasty for an invasive tumor could potentially upgrade the stage of an ovarian cancer, thereby affecting the treatment and the prognosis.

Since 2013, we have been performing intraoperative ultrasound scans in selected young patients with benign ovarian tumors (by pre-operative ultrasound or magnetic resonance imaging). Regarding ovarian preservation, we compared the patients who underwent intraoperative ultrasound and those who did not.

## Methods

### Patient Selection

Most of the women of reproductive age who undergo intraoperative ultrasound for benign ovarian tumors at the A.C. Camargo Cancer Center (Brazil) are diagnosed with a teratoma. Therefore, a retrospective search was conducted in the archives of the center to identify cases involving *teratoma* and *intraoperative ultrasound* that were treated between November 2007 and June 2017. The eligible female patients were aged between 14 and 40 years, and their surgeries were performed in the Gynecologic Oncology Department of our center. Patients with a history of gynecologic cancer, non-gynecologic tumors metastasizing to the ovaries, and mixed tumors were excluded. The women who met these criteria (*n* = 36) were divided into two groups: those who underwent intraoperative ultrasound (group A, *n* = 25) and those who did not (group B, *n* = 11). The primary endpoint was ovarian sparing surgery (oophoroplasty), and the present study was approved by the Ethics in Research Committee of our institution.

### Surgery

Minimally-invasive surgery was performed for most patients, with open laparoscopy used to gain peritoneal access. Briefly, a puncture of ∼ 1 cm in diameter was made in the umbilical or supraumbilical area, and dissection of the abdominal layer was performed under direct vision until the peritoneal cavity was accessed. Additional punctures with diameters of 0.5 cm to 1 cm were made in the bilateral lower quadrants and suprapubic region under direct vision as well. Pneumoperitoneum was established with carbon dioxide at a pressure setting of 12 mmHg. A complete pelvic examination was performed to exclude malignant disease. At the surgeon's discretion, an oophorectomy or oophoroplasty was subsequently performed. All cysts were removed from the abdominal cavity with endobags (Medtronic, Minneapolis, MN, US). Four patients had laparotomy as the primary access.

### Intraoperative Ultrasound

**Fig. 1:**
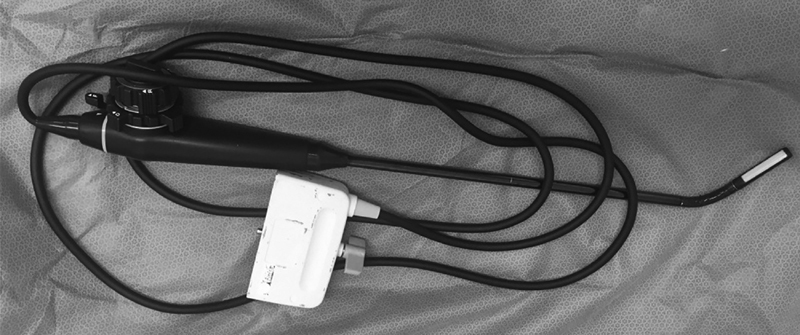
Ultrasound laparoscopic probe.

For the patients in group A, a NemioXG (Toshiba, Minato, Tokyo, Japan) device with a laparoscopic probe (5.0–10.0 MHz laparoscopic transducer for SSA-370A) was used. For the patients who underwent laparotomy, a linear probe (8.0–14.0 MHz linear transducer) was used. The ultrasound laparoscopic probe (Fig. 1) was inserted through a 12-mm trocar located in the suprapubic or left inferior lower quadrant. Conventional gray scale sonography was performed by the surgeon, and normal ovarian parenchyma, solid components, and septa were evaluated.

### Statistical Analysis

The STATA (STATA Corp., College Station, TX, US) software, version 10.0, was used to perform all of the statistical analyses. Frequency distribution was used to describe the categorical variables, and measures of central tendency and variability were used for the numerical variables. The non-parametric Mann-Whitney U-test was applied to verify the association of the numerical variables according to patient group. The Fisher Exact test was used to compare the categorical variables according to each group in 2 × 2 tables when at least 1 expected frequency was lower than 5. The Shapiro-Wilk test was used to verify the normality of the numerical data. A 5% significance level was established for all of the statistical tests.

## Results

A total of 82 eligible cases were identified, among which 36 cases fulfilled our inclusion criteria. In 25 of these cases, intraoperative ultrasound was performed. The patients in groups A and B were similar regarding arterial hypertension, diabetes, smoking history, and body mass index (BMI) (Table 1). The overall mean age of the two groups was 27.7 years, with the patients in group A being younger than those in group B by only a marginally significant difference (*p* = 0.052). Nulliparity was higher in group A (84%) compared with group B (50%), although the difference was not statistically significant (*p* = 0.081).

**Table 1:** Patient characteristics before surgery

Variable	Category	Intraoperative ultrasound: group A (yes) group B (no)		*p*-value
Age (years)	Number	25	11	**0.052**
	Range	15–39	15–40	
	Median	26.0	35.0	
	Mean (standard deviation)	26.0 (7.9)	31.7 (8.4)	
Symptoms, number (%)	No	20 (80.0)	9 (81.8)	0.999*
	Yes	5 (20.0)	2 (18.2)	
Arterial hypertension, number (%)	No	25 (100.0)	10 (90.9)	Not able to be evaluated
	Yes	0 (0.0)	1 (9.1)	
Diabetes, number (%)	No	25 (100.0)	11 (100.0)	Not able to be evaluated
	Yes	0 (0.0)	0 (0.0)	
Smoking, number (%)	No	25 (100.0)	11 (100.0)	Not able to be evaluated
	Yes	0 (0.0)	0 (0.0)	
Previous ovarian surgery, number (%)	No	23 (92.0)	10 (90.9)	0.999*
	Yes	2 (8.0)	1 (9.1)	
Menstrual cycle, number (%)	Eumenorrheic	24 (96.0)	10 (90.9)	0.524*
	Dysmenorrheic	1 (4.0)	1 (9.1)	
Body mass index	Number	25	11	0.450
	Range	16.5–31.2	17.2–37.3	
	Median	22.3	23.4	
	Mean (standard deviaiton)	22.5 (3.3)	24.1 (5.5)	
Pregnancy	0	19 (76.0)	5 (50.0)	0.227*
	1+	6 (24.0)	5 (50.0)	
Parity	0	21 (84.0)	5 (50.0)	0.081*
	1+	4 (16.0)	5 (50.0)	
Miscarriage	0	23 (92.0)	9 (90.0)	0.999
	1+	2 (8.0)	1 (10.0)	
Type of delivery, number (%)	Vaginal	0 (0.0)	2 (40.0)	0.444
	Cesarean-section	4 (100.0)	3 (60.0)	

Notes: *p*-value obtained by the Mann-Whitney U-test; *p-value obtained by the Fisher exact test.

Most of the patients in both groups were asymptomatic (80.0% and 81.8% in groups A and B respectively). A laparoscopic approach was used in 96% of the surgeries in group A, and in 72.7% of the surgeries in group B. Tumor size was similar between the two groups, ranging from 1 cm to 11 cm in group A from 1.3 cm to 10 cm in group B (Table 2).

**Table 2:** Clinical variables of surgeries with and without intraoperative ultrasound

Variable	Category	Intraoperative ultrasound: group A (yes) group B (no)		*p*-value
Ovarian side, number (%)	Right	9 (36.0)	7 (63.6)	Not able to be evaluated
	Left	11 (44.0)	4 (36.4)	
	Both	5 (20.0)	0 (0.0)	
Surgical access, number (%)	Laparoscopy	24 (96.0)	8 (72.7)	0.076
	Laparotomy	1 (4.0)	3 (27.3)	
Histology, number (%)	Mature teratoma	17 (68.0)	11 (100.0)	Not able to be evaluated
	Bilateral mature teratoma	3 (12.0)	0 (0.0)	
	Mature teratoma and endometrioma	1 (4.0)	0 (0.0)	
	Endometrioma	2 (8.0)	0 (0.0)	
	Fibroma	2 (8.0)	0 (0.0)	
Surgery performed, number (%)	Oophorectomy	6 (24.0)	10 (90.9)	< 0.001
	Oophoroplasty	19 (76.0)	1 (9.1)	
Tumor diameter (cm), number (%)	Number	25	10	0.594*
	Range	1–11	1.3–10	
	Median	4.0	5.5	
	Mean (standard deviation)	5.2 (3.0)	5.6 (2.6)	
Major complications, number (%)	No	25 (100.0)	11 (100.0)	Not able to be evaluated
	Yes	0 (0.0)	0 (0.0)	
Menstrual cycle postsurgery, number (%)	Eumenorrheic Dysmenorrheic	21 (84.0) 1 (4.0)	7 (63.6) 0 (0.0)	Not able to be evaluated
	Amenorrheic	0 (0.0)	1 (9.1)	
	Not known	3 (12.0)	3 (27.3)	

Notes: *p*-value obtained by the Mann-Whitney U-test; *p-value obtained by the Fisher exact test.

Regarding the primary endpoint, a more conservative surgery was achieved with the use of the intraoperative ultrasound, and the difference between groups A and B was statistically significant (*p* < 0.001). For example, 76% of the patients in group A underwent oophoroplasty, while only 9.1% of the patients in group B were submitted to the conservative management of the ovarian parenchyma. Bilateral oophorectomy was not performed for any of the patients in the present study, and there were no major surgical complications in either of the groups. Tumor histology confirmed mature teratomas for all of the cases in group B and for most of the cases in group A.

## Discussion

Because the present study is retrospective, with all of its implied limitations, it could be argued that a special effort was made in order to achieve ovarian preservation in some patients. However, we selected patients under 40 years old to avoid this issue. Despite the fact that there were younger women in group A, this difference was not significant.

When a young woman presents with an ovarian cyst, a surgeon must decide whether or not to operate. For this decision, it is imperative to evaluate the risk of malignancy and to consider the possibility of performing ovarian sparing surgery in benign cases. For the latter, signs of a benign lesion and the extent of normal ovarian tissue that can be spared are key. Papillae within a cyst can be indicative of an invasive and borderline tumor, while sonography can characterize mature teratomas, endometriomas, ovarian fibromas, and serous cystadenocarcinomas.^6^ ^7^ ^8^ ^9^ An intraoperative scan can detect transvaginal unilocular cysts that present solid components.^10^

In 2012, we reported two cases of ovarian cysts that were examined by intraoperative vaginal ultrasound. For this procedure, a standard vaginal probe was inserted in the pelvic cavity that was filled with 0.9% saline solution. The presence of the saline solution created an interface with the surgical instruments that facilitated the identification of normal ovarian parenchyma.^11^ Since then, our institution has acquired a laparoscopic probe that enables us to perform intraoperative ultrasound directly on a target organ, thereby rendering our prior technique obsolete.

Yang et al^12^ compared the accuracy of laparoscopic ultrasonography for the characterization of adnexal masses to transvaginal sonography that was performed one day before the surgery. The accuracy of laparoscopic ultrasonography in the characterization of adnexal masses was of 83.8%, and that of transvaginal sonography was of 73.5% (*p* < 0.05). Moreover, laparoscopic sonography showed greater morphological detail, it enabled a more precise and specific characterization of the adnexal masses, and it detected additional adnexal lesions that were not evident by preoperative transvaginal sonography. Laparoscopic ultrasonography was also found to be superior in revealing the presence of residual ovarian tissue on the pathologically-affected side compared with transvaginal sonography (76.5% versus 59.4% respectively). In our practice, we believe intraoperative ultrasonography was superior in the evaluation of regular ovarian tissue, even compared with magnetic resonance.

Laparoscopic surgery is widely accepted as a standard treatment for benign ovarian cysts.^13^ ^14^ In our series, most of the patients were managed by laparoscopy, and the tumor diameters between groups A and B did not differ significantly. It has been demonstrated that laparoscopy has a high diagnostic accuracy in the evaluation of benign ovarian masses.^14^

**Fig. 2:**
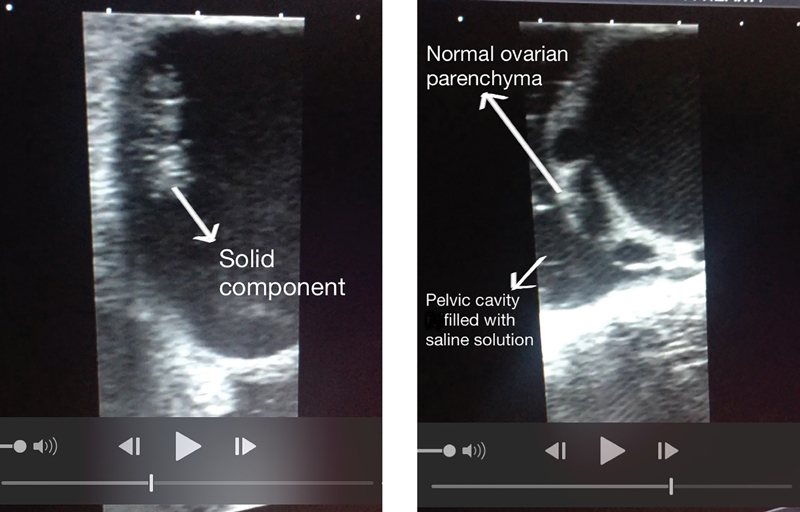
A solid component and normal ovarian parenchyma present in the same cyst. The saline solution served as an efficient image interface.

Large ovarian cysts have been shown to have a higher incidence of malignancy.^15^ After the risk of malignancy has been excluded, the extent of normal ovarian parenchyma that is present is evaluated. In addition to standard sonography exams, the presence of an “ovarian crescent sign” is an indicator of a benign lesion, and it is useful to guide the surgeon during ovarian sparing surgery (Fig. 2). Visualization of healthy ovarian tissue does not require a high level of ultrasound skill.^16^

Usually, an incision is made on the mesial side of the ovary, and a cystectomy is performed, thereby sparing healthy ovarian tissue.^17^ ^18^ ^19^

In our series, most patients had mature teratomas, while some presented with endometrioma and fibroma. Most of the patients were managed clinically and were asymptomatic. However, a few cases were managed surgically for the aforementioned reasons. For these patients who desire to preserve ovarian function and/or fertility, it was important to offer the possibility of performing an ovarian parenchyma sparing procedure, and intraoperative ultrasound played a significant role in that.

## Conclusion

The use of intraoperative ultrasound resulted in more conservative surgeries for the resection of benign ovarian tumors at our center.
